# Activating mutations in *CTNNB1* in aldosterone producing adenomas

**DOI:** 10.1038/srep19546

**Published:** 2016-01-27

**Authors:** Tobias Åkerström, Rajani Maharjan, Holger Sven Willenberg, Kenko Cupisti, Julian Ip, Ana Moser, Peter Stålberg, Bruce Robinson, K. Alexander Iwen, Henning Dralle, Martin K. Walz, Hendrik Lehnert, Stan Sidhu, Celso Gomez-Sanchez, Per Hellman, Peyman Björklund

**Affiliations:** 1 Department of Surgical Sciences, Uppsala University, Uppsala, Sweden; 2 Department of Endocrinology and Metabolism, Rostock University Medical Center, Germany; 3 General, Visceral and Pediatric Surgery University Hospital Düsseldorf, Düsseldorf, Germany; 4 University of Sydney, Endocrine Surgical Unit and Cancer Genetics, Hormones and Cancer Group, Kolling Institute of Medical Research, Royal North Shore Hospital, Sydney, Australia; 5 Department of Medicine I, University of Lübeck, University Hospital Schleswig-Holstein, Lübeck, Germany; 6 Department of General, Visceral and Vascular Surgery, University Hospital, University of Halle-Wittenberg, Halle/Saale, Germany. Sweden; 7 Klinik für Chirurgie und Zentrum für Minimal Invasive Chirurgie, Kliniken Essen-Mitte, Essen, Germany; 8 Endocrine Section, Department of Medicine, G.V. (Sonny) Montgomery VA Medical Center and University of Mississippi Medical Center, Jackson, USA

## Abstract

Primary aldosteronism (PA) is the most common cause of secondary hypertension with a prevalence of 5–10% in unreferred hypertensive patients. Aldosterone producing adenomas (APAs) constitute a large proportion of PA cases and represent a surgically correctable form of the disease. The WNT signaling pathway is activated in APAs. In other tumors, a frequent cause of aberrant WNT signaling is mutation in the *CTNNB1* gene coding for β-catenin. Our objective was to screen for *CTNNB1* mutations in a well-characterized cohort of 198 APAs. Somatic *CTNNB1* mutations were detected in 5.1% of the tumors, occurring mutually exclusive from mutations in *KCNJ5, ATP1A1, ATP2B3* and *CACNA1D*. All of the observed mutations altered serine/threonine residues in the GSK3β binding domain in exon 3. The mutations were associated with stabilized β-catenin and increased AXIN2 expression, suggesting activation of WNT signaling. By CYP11B2 mRNA expression, CYP11B2 protein expression, and direct measurement of aldosterone in tumor tissue, we confirmed the ability for aldosterone production. This report provides compelling evidence that aberrant WNT signaling caused by mutations in *CTNNB1* occur in APAs. This also suggests that other mechanisms that constitutively activate the WNT pathway may be important in APA formation.

Primary aldosteronism (PA) is characterized by autonomous aldosterone production with increased aldosterone to renin ratio^1^. The increased level of aldosterone leads to abnormal retention of sodium and water, and potassium excretion, causing hypervolemic hypertension, and in more severe cases, hypokalemia^2^. Once thought to be a rare disease, PA is now being recognized as the most common cause of secondary hypertension with a prevalence of 5–10% in hypertensive individuals and up to 20% in therapy-resistant hypertension^3^,^4^,^5^,^6^. Most commonly, PA is either caused by unilateral aldosterone producing adenoma (APA) or bilateral adrenal hyperplasia (BAH)^3^,^4^. Unilateral APAs represent about 30% of PA cases^3^,^4^,^5^. Dominant unilateral aldosterone production caused by either APA or asymmetrical aldosterone release in the context of BAH can be cured by surgery, while patients with symmetrical BAH should be recommended mineralocorticoid receptor antagonists^7^. The importance of detecting PA patients among hypertensive individuals is not only due to the possibility for specific treatment and potential cure, but also because PA is an independent risk factor for increased cardiovascular morbidity, caused by excess aldosterone, independent of blood pressure^8^,^9^,^10^,^11^.

The WNT signaling pathway is important for normal development and maintenance of the adrenal cortex, and more specifically, the zona glomerulosa (ZG) within the cortex^12^,^13^. Exon 3 of the *CTNNB1* gene (encoding β-catenin), contains specific serine and threonine residues that, when phosphorylated, mark β-catenin for degradation^14^,^15^. Mutations affecting these amino acids, or deletions of exon 3, subsequently preclude phosphorylation of β-catenin, leading to aberrant activation of WNT signaling^16^,^17^,^18^,^19^,^20^.

Active WNT signaling has been observed in APAs^21^,^22^,^23^. The Secreted Frizzled-related protein II (SFRP2) is a negative regulator of the WNT pathway^24^, and its down-regulation has been proposed as one of the mechanisms for this increase in WNT signaling^21^. Mice with abnormal WNT signaling, due to alterations in exon 3 of the *CTNNB1* gene, develop hyperaldosteronism and adrenocortical tumors^23^,^25^. Mutations in *CTNNB1* also cause increased, abnormal WNT activation in human adrenocortical tumors^26^,^27^. Two independent exome sequencing experiments have demonstrated *CTNNB1* mutations in two APAs^28^,^29^. The aim of this study was to determine the prevalence of *CTNNB1* mutations in APAs.

## Results

### Mutation status of *CTNNB1*

The included APA samples exhibited mutations in *KCNJ5* (n = 92, 46.5%), *ATP1A1* (n = 6, 3.0%), *ATP2B3* (n = 3, 1.5%), *CACNA1D* (n = 3, 1.5%), while 94 (47.5%) did not harbor mutations in known genes^30^,^31^. Somatic missense mutations in *CTNNB1* were observed in 10 APAs (5.1% in the total cohort and in 10.6% of APAs without known mutations). No large deletions of exon 3 were observed in investigated non-mutated tumors (n = 7, Supplementary Fig. S1 online). The prevalence of *CTNNB1* mutations was similar among different national cohorts (Supplementary Table S1 online). The detected mutations all occurred in conserved serine/threonine residues in exon 3 (Supplementary Fig. S2, Supplementary Table S2 online). The mutations were absent from constitutional DNA in all available specimens (n = 7), confirming their somatic nature. All mutations in *CTNNB1* occurred mutually exclusive to *KCNJ5, ATP1A1, ATP2B3* and *CACNA1D* (p = 1.47e^-14^, Fisher’s exact test).

### Aldosterone production in *CTNNB1* mutant tumors

**Figure 1:**
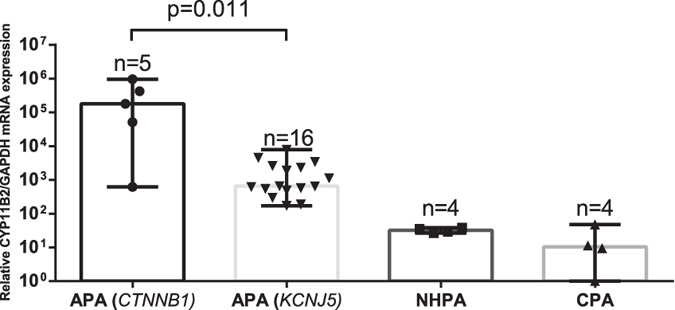
CYP11B2 expression in APAs. Relative mRNA expression of CYP11B2 in APAs with *CTNNB1* mutation (n = 5) compared to APAs with *KCNJ5* mutation (n = 16), NHPA (n = 4) and CPA (n = 4) using the 2^(−ΔΔCT)^ method. GAPDH was used as the reference gene. Values indicate median with range. Y-axis shows logarithmic values. NHPA = Non-hormone producing adenoma. CPA = Cortisol producing adenoma. Statistical analysis using Mann -Whitney U-test.

**Figure 2:**
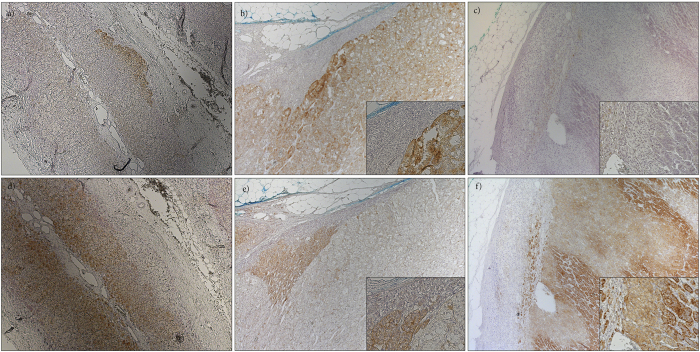
CYP11B1/B2 protein expression. Immunohistochemical analysis of CYP11B1/B2 expression. (**a**) CYP11B2 staining of normal adrenal gland. (**b**) CYP11B2 staining of a tumor with *CTNNB1* mutation displaying diffuse expression. (**c**) CYP11B2 staining of a tumor with *CTNNB1* mutation displaying weak heterogeneous expression. (**d**) CYP11B1 staining of normal adrenal gland. (**e**) CYP11B1 staining of a tumor with *CTNNB1* mutation displaying weak heterogeneous expression. (**f**) CYP11B1 staining of a tumor with *CTNNB1* mutation displaying diffuse expression.

**Figure 3:**
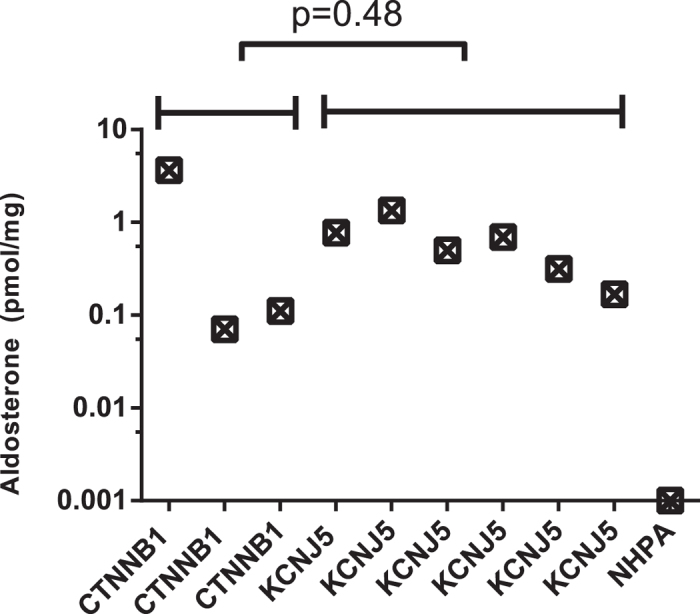
Aldosterone concentration in tumor lysate (pmol/mg). Aldosterone concentration in tumor lysates using a chemiluminescent immunoassay. Statistical analysis using Mann -Whitney U-test.

We analyzed CYP11B2 mRNA expression by semi-quantitative RT-PCR in *CTNNB1* mutated and *KCNJ5* mutated tumors, as well as in NHPAs and CPAs. The investigated *CTNNB1* mutants displayed higher CYP11B2 expression than NHPAs, CPAs and also significantly higher than tumors with *KCNJ5* mutations (p = 0.011) (Fig. 1). CYP11B2 protein expression was analyzed by IHC using a mouse monoclonal antibody. Staining of normal adrenal tissue revealed heterogeneous expression selective for ZG (Fig. 2a). All investigated *CTNNB1* mutated tumors (n = 8) exhibited expression of CYP11B2. Four had diffuse expression throughout the tumor (Fig. 2b), while four displayed limited heterogeneous staining (Fig. 2c). To further confirm the ability of *CTNNB1* mutated tumors to produce aldosterone, aldosterone concentration were directly measured in tumor tissue lysates utilizing an automated immunoassay. The investigated *CTNNB1* mutated APA lysates (n = 3) contained aldosterone at similar levels as *KCNJ5* mutated tumor lysates (p = 0.48, Fig. 3).

### Histologic appearance and CYP11B1/B2 protein expression in APAs with *CTNNB1* and *KCNJ5* mutation

**Figure 4:**
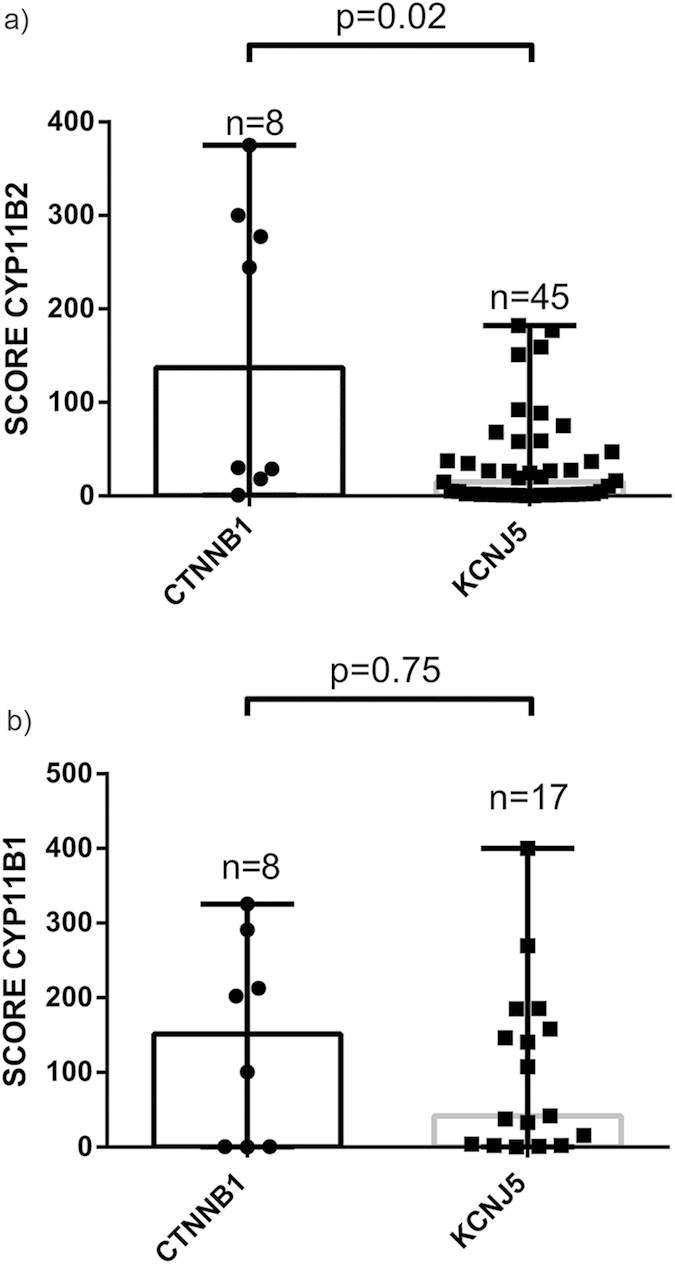
Comparison of CYP11B1/B2 expression score in APA. Immunohistochemical expression score of CYP11B2 and CYP11B1 in tumors with *CTNNB1* and *KCNJ5* mutation.Y-axis display the expression score of CYP11B2, calculated using a modified H-score. (**a**) CYP11B2. (**b**) CYP11B1. Median with range. Statistical analysis using Mann-Whitney U-test.

Histologic examination of *CTNNB1* mutated tumors did not reveal any specific cell morphology (Supplementary Table S3 online). CYP11B1 antibody specificity was confirmed in normal adrenal tissue (Fig. 2d). Staining of eight *CTNNB1* mutated tumors revealed two distinct subgroups. In tumors with high CYP11B2 expression the CYP11B1 expression was also low (Fig. 2b,e). Similarly, in those with low CYP11B2 expression the CYP11B1 staining was strong (Fig. 2c,f). Comparing with *KCNJ5* mutated APAs, no difference in CYP11B1 expression levels were observed, but a significantly higher CYP11B2 expression in *CTNNB1* mutated tumors were observed (p = 0.02, Fig. 4a,b).

### β-catenin accumulation and activated WNT signaling in tumors with *CTNNB1* mutation

**Figure 5:**
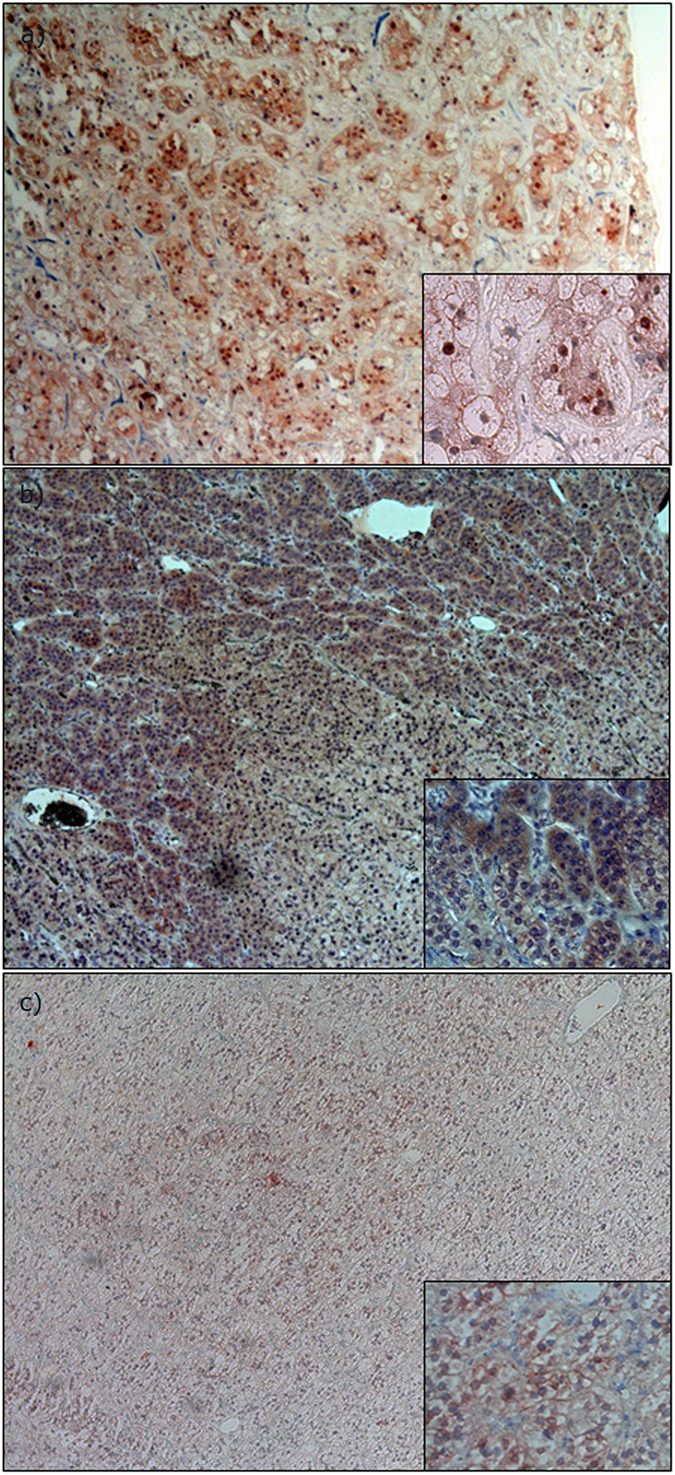
Immunohistochemical expression analysis of β-catenin. (**a**) APA with *CTNNB1* mutation displaying nuclear and cytoplasmic β-catenin expression. (**b**) APA with *CTNNB1* mutation displaying heterogeneous cytoplasmic expression. (**c**) APA with *CTNNB1* mutation with membranous and nuclear expression.

**Figure 6:**
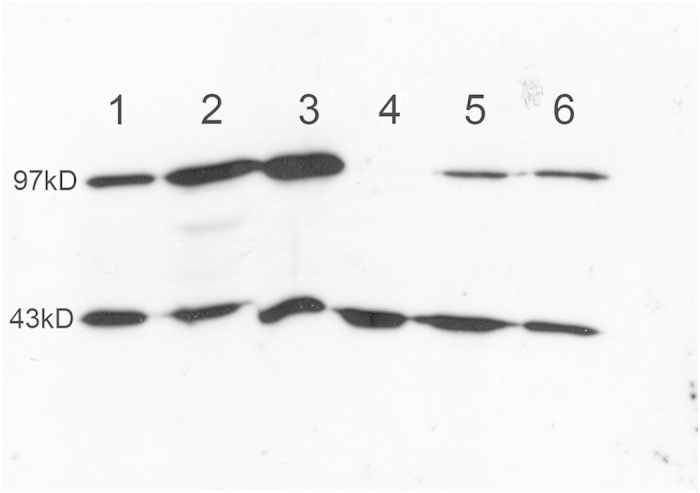
Western blot analysis using an anti-active β-catenin antibody. Western blot analysis on three APAs with *CTNNB1* mutation (1–3), one APA without known mutation (4) and two APAs with *KCNJ5* mutation (5–6). Top row shows signal for active β-catenin (97 kD), lower row shows actin signal (43 kD).

**Figure 7:**
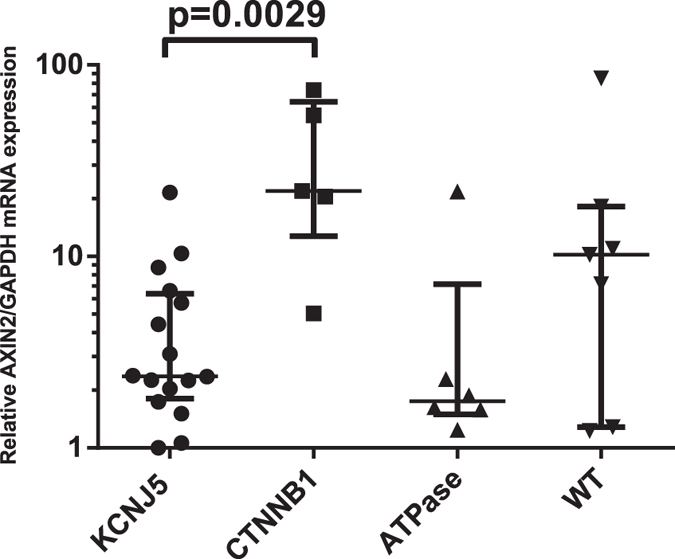
AXIN2 mRNA expression. Relative mRNA expression of AXIN2 in APAs with *CTNNB1* mutation (n = 5) compared with APAs with *KCNJ5* mutation (n = 16), APAs with *ATP1A1* and *ATP2B3* mutation (n = 6), and seven APAs without known mutation, using the 2^(−ΔΔCT)^ method. GAPDH was used as the reference gene. Values indicate median with interquartile range. Statistical analysis using Mann -Whitney U-test.

All investigated APAs with *CTNNB1* mutation displayed cytoplasmic and/or nuclear β-catenin staining (n = 8, Fig. 5), and displayed high levels of active β-catenin by western blot (n = 3, Fig. 6). No apparent nuclear staining was observed in tumors without *CTNNB1* mutation, although 11/24 (45.8%) displayed cytoplasmic staining (H-score ≥50, Supplementary Table S4 and Supplementary Fig. S3 online). In the tumors without *CTNNB1* mutation, β-catenin expression correlated with ZG-like cell content (r^2^ = 0.61, p < 0.0001, Supplementary Fig. S4 online). We did not observe any genotype-expression correlation as tumors with *KCNJ5* mutation and ZG-like cell content displayed cytoplasmic expression of β-catenin (Supplementary Fig. S3 online). Western blot in one tumor with *CTNNB1* mutation (p.Ser45Pro) and four without *CTNNB1* mutation, validated antibody specificity for β-catenin (Supplementary Fig. S5 online). To analyze downstream targets of β-catenin, AXIN2 mRNA expression was measured. A significantly higher expression of AXIN2 in tumors with *CTNNB1* mutation compared to those with *KCNJ5* mutation was observed (p = 0.0029, Fig. 7). In five tumors without known mutations we observed high levels of AXIN2 suggesting active WNT signaling.

### Clinical characteristics of patients with tumors harboring *CTNNB1* mutation

The clinical data of the patients with tumors harboring *CTNNB1* mutation are summarized in Supplementary Table S5 online. *CTNNB1* mutated APAs were more often found in female patients (60%). There were no significant differences in preoperative aldosterone levels, tumor size, or age at surgery in patients with tumors harboring *CTNNB1* mutation compared to those with tumors harboring *KCNJ5* mutation (Supplementary Table S5 online). However a significant difference in size between the tumors with *CTNNB1* mutation and tumors without known mutation was observed, median 24 mm (range 10–45) vs median 14 mm (range: 3–45, mean value 14.7 mm ± 7.9SD) (p = 0.01).

## Discussion

WNT signaling is essential for normal development and maintenance of the adrenal cortex^13^. In the canonical WNT signaling pathway, absence of WNT ligands induces formation of a complex consisting of Dishevelled, AXIN, APC, CKI and GSK3β which binds and phosphorylates β-catenin, leading to its ubiquitination and subsequent degradation by proteasomes^32^. In contrast, WNT ligand binding to the frizzled and LRP co-receptors causes sequestering of the destruction complex at the cytoplasmic membrane, leaving it unable to phosphorylate. This leads to β-catenin accumulation, translocation to the nucleus, and altered transcription of downstream targets, including: T-cell factor/lymphoid enhancer factor (TCF/LEF-1), CYP21, the Angiotensin I receptor and CYP11B2^21^,^33^,^34^. β-catenin activation in the adrenal gland is normally restricted to cells of the ZG^22^. Stabilizing mutations in the *CTNNB1* gene increase the activity of the finely tuned WNT signaling pathway, leading to tumor formation^16^. Previous reports have shown that mutations in β-catenin occur in a small proportion of APAs^26^,^28^,^29^,^35^. Yet contradictorily, two different studies sequencing APAs did not detect any *CTNNB1* mutations^21^,^22^. Here we performed *CTNNB1* sequencing in a large cohort of clinically and molecularly diagnosed APAs. We detected *CTNNB1* mutations in 5.1% of cases, all affecting previously described mutation “hotspot” amino acids p.Thr41 and p.Ser45. All *CTNNB1* mutations occurred mutually exclusive to *KCNJ5*, *ATP1A1*, *ATP2B3* and *CACNA1D* mutated tumors, implying that aberrant WNT activation may be enough for APA formation as indicated in mouse models^23^. Tumors with *CTNNB1* mutations were associated with relatively large adenomas, and were found both in female and male patients, however with a small overrepresentation in females.

To analyze the effect on WNT signaling, we performed β-catenin IHC and quantitative PCR on AXIN2. We observed nuclear and/or cytoplasmic β-catenin staining in tumors with *CTNNB1* mutation, indicating aberrant, increased protein accumulation. We also noted APAs without *CTNNB1* mutation having cytoplasmic β-catenin expression and also those with relatively high mRNA expression of AXIN2. This suggests that WNT signaling is activated in a large subgroup of APAs and independent of genetic background^22^, raising the question if other genetic aberrations in the WNT pathway may be involved in development of APAs.

CYP11B2 and CYP11B1 are highly homologous enzymes expressed in ZF and ZG respectively. CYP11B1 converts 11-deoxycortisol to cortisol, whereas CYP11B2 converts deoxycorticosterone successively to corticosterone, 18-OH-corticosterone and finally aldosterone. CYP11B2 expression indicates aldosterone production, and most APAs display expression, although in some cases heterogeneously^36^,^37^. Utilizing specific mRNA probes and primers for CYP11B2^38^, we found expression of CYP11B2 mRNA in tumors with *CTNNB1* mutations indicating ability to produce aldosterone. IHC utilizing an antibody against CYP11B2 showed high specificity for ZG. We verified expression of CYP11B2 in all *CTNNB1* tumors. Four *CTNNB1* mutated tumors demonstrated diffuse expression, while four tumors exhibited heterogeneous and relatively weak staining, similar to the pattern seen in many of the tumors with *KCNJ5* mutation^36^,^37^. To further confirm aldosterone production, we measured aldosterone concentration in tumor lysates using a chemiluminescent immunoassay. The investigated mutants contained aldosterone, and at similar levels to other APAs with *KCNJ5* mutations indicating an active aldosterone production. Measuring aldosterone in tumor tissue may provide a diagnostic tool to discriminate functional aldosterone producing nodules from non-functional and with introduction of faster assays, could even be employed in the operating theatres.

APAs may contain a varying degree of ZG-like cells and ZF-like cells^39^. Some reports indicate that this phenotype depends on underlying genetic alteration^29^,^37^,^40^, whereas others do not^41^. Also, a difference in the protein expression pattern of CYP11B2/B1 has been observed between the different mutants^36^,^37^. By histologic examination, we observed a variable histologic appearance in *CTNNB1* mutated tumors. Interestingly, we observed two subgroups of tumors; one with diffuse CYP11B2 expression with concomitantly low CYP11B1 expression, and one with low CYP11B2 and high CYP11B1 expressions. Perhaps suggesting that these mutations mainly promote proliferation.

*CTNNB1* mutations occur in cortisol producing adenomas (CPA). Recently, mutations in the catalytic subunit of Protein kinase A (PKA) were identified and shown to occur mutually exclusive to *CTNNB1* mutations^42^,^43^,^44^,^45^. Activating mutations in PKA lead to a constitutively activated cAMP signaling, causing increased cortisol production and likely tumor formation. Expression analysis revealed increased expression of genes involved in biosynthesis and metabolism of steroids in tumors with *PRKACA* mutation^42^. This suggests two pathways for CPA formation: either alterations in cAMP signaling with increased steroidogenesis, or other mutations mainly stimulating proliferation. In APAs, most mutations observed affect the membrane potential and increase the likelihood of depolarization. These tumors harbor few mutations per megabase, and seldom have CNV alterations^28^. In contrast, many APAs without known mutation have CNV alterations^28^, suggesting different pathways for APA development, similar to CPAs. We propose that *CTNNB1* mutations belong to a group of alterations mainly affecting proliferation rate. The low prevalence of mutations in APAs compared to NHPAs or CPAs could reflect the low number of ZG cells expressing CYP11B2 in normal adrenal gland.

It is unknown if carcinomas of the adrenal cortex evolve in an adenoma-to-carcinoma sequence^46^. Evidence in favor of this hypothesis comes from experiments using β-catenin mutated mice that develop benign tumors that can progress to malignancy, suggesting requirement of additional genetic alterations^23^,^25^. This is in line with the multistep progression model seen in patients with familial adenomatous polyposis^47^. Moreover, signs of carcinomas inside benign adrenal masses have been described^48^,^49^,^50^. However, most APAs rarely increase in size and aldosterone producing carcinomas are exceedingly rare^7^. If adenoma to carcinoma sequences does occur, one could speculate that patients with tumors harboring *CTNNB1* mutation have a small but increased risk of malignant transformation. Another interesting observation, coming from the β-catenin stabilized mouse model is that up to 10 months of age the mice produce aldosterone and show no signs of corticosterone production. Despite this, 17-month-old mice exhibit a trend towards lower aldosterone synthesis and increased corticosterone production, suggesting that the level of WNT activity determine the functional characteristics of the lesion^23^. This could also explain why mutations in *CTNNB1* are less frequent in APAs.

In conclusion, we observed *CTNNB1* mutations in a subset of APAs with aberrant β-catenin accumulation. Tumors harboring these mutations have a variable histological and CYP11B2/B1 expression pattern, produce aldosterone, and show similar clinical characteristics as patients with tumors harboring *KCNJ5* mutation. We speculate that *CTNNB1* mutations lead to a proliferative advantage in the adrenal cell it occurs in. Because of the higher frequency of WNT activation in APAs than *CTNNB1* mutations, future investigations of alterations in other members of the WNT pathway are warranted.

## Materials and Methods

### Tissues

Adrenocortical tumor tissues were collected from six different centers. According to routine protocols at the centers, a diagnosis of APA was established by these criteria: an elevated aldosterone/renin ratio together with positive confirmatory tests and lateralization studies by CT, MRI and/or adrenal vein sampling, and a postoperative cure or considerable improvement. Patients with cortisol producing adenomas (CPA) had elevated serum cortisol, failure to suppress cortisol with dexamethasone, normal aldosterone levels and all were postoperatively cured. Non-hormone producing adenomas (NHPA) were detected *en passant* by abdominal CT scan in biochemically normal and asymptomatic patients, and were removed due to large size or malignant features on CT.

### DNA and RNA extraction

DNA and RNA from frozen tissues were extracted using Allprep DNA/RNA kit, (Qiagen, Hilden, Germany) or FFPE tissue using AllPrep DNA/RNA FFPE Kit (Qiagen, Hilden, Germany) and from blood using DNeasy Blood & Tissue Kit (Qiagen, Hilden, Germany). Synthesis of cDNA was performed using the First-Strand cDNA Synthesis kit according to the manufacturer’s instructions (Fermentas, Thermo Fisher Scientific, Waltham, USA).

### PCR amplification and DNA sequencing

Exon 3 of the *CTNNB1* gene was amplified by PCR amplification, using primers: FW: 5′-TGTCTTTCAGATTTGACTTTATTT, RW: 5′-TCAAAACTGCATTCTGACTTTCA followed by a nested reaction with FW2: 5′-CAATGGGTCATATCACAGATTCTT, then Sanger sequenced. Traces were analyzed and compared to the *CTNNB1* reference sequence NP_001895.1. Observed mutations were validated in an independent PCR reaction. cDNA was sequenced using mRNA specific primers, cDNA FW: 5′-GCTACTCAAGCTGATTTGATG and RW: 5′-CAGGACTTGGGAGGTATCC. Large deletions affecting exon 3 of the *CTNNB1* gene were investigated by RT-PCR amplification using mRNA specific primers, FW primer: 5′-GAAGGTCTGAGGAGCAGCTTC and RW: 5′-GACATTAGATGAGGGCATGC. The obtained amplicons were run on a 2% agarose gel to visualize size of the products. All tumors had previously been sequenced for *KCNJ5*, *ATP1A1*, *ATP2B3* and *CACNA1D* mutations^31^.

### Histology examination and immunohistochemistry

Tumors were evaluated to determine their zona glomerulosa (ZG) and zona fasciculata (ZF) cell content. Cells were classified as ZG-like if they had a high nuclear to cytoplasmic ratio and formed circular cell clusters. Cells were classified as ZF-like if they were lipid-rich with a high cytoplasm to nuclear ratio and a more uniform sponge-like phenotype^39^. IHC for β-catenin was conducted on eight tumors with *CTNNB1* mutation and 24 tumors without *CTNNB1* mutation, using an anti-β-catenin goat polyclonal antibody (Santa Cruz Biotechnology, Inc., Santa Cruz, CA, USA; catalog no. sc-1496, dilution 1:5) as previously described^18^. Staining of CYP11B1 and CYP11B2 were conducted using rat and mouse monoclonal antibodies respectively^51^. Briefly, 5 μm sections were deparaffinized in xylene and rehydrated through graded ethanol. Endogenous peroxidases were inhibited by incubation in hydrogen peroxide solution. Antigen retrieval using 10 mM sodium citrate (pH6.0) was used. Sections were then incubated with 10% normal goat serum/horse serum after which the primary antibody was added (CYP11B1, 1:100 and CYP11B2, 1:250) overnight. For detection, biotinylated HRP conjugated goat anti-rat or horse anti-mouse secondary antibodies were used, 1:200 (Vector Laboratories, Burlingame, USA). The sections were developed with DAB (Vector Laboratories, Burlingame, USA) and counterstained with hematoxilin. Two researchers, unaware of the mutational status, conducted comparison of expression. A scoring system using a modified H-score was applied^52^. Five 20× fields, and the entire sample were scored on intensity *i* (0 = negative, 1 = weak but detectable, 2 = distinct, 3 = strong), and percentage of stained cells *P*_*i*_. A mean value of these six measurements was used in the formula ∑ = *P*_*i*_(*i *+ 1). Images were acquired using a Zeiss Observed Z1 microscope (Zeiss, Oberkochen, Germany) and imported using the AxioVision SE64 4.9.1 software (Zeiss, Oberkochen, Germany).

### Western blot

To verify specificity of the β-catenin antibody used in the IHC experiment, western blot analysis was performed on one *CTNNB1* mutated APA (p.Ser45Pro) and four APAs without *CTNNB1* mutation (Santa Cruz-1496; dilution 1:1000), with anti-actin (Santa Cruz-1616) as loading control. Western blot was also performed on three tumors with *CTNNB1* mutation, two with *KCNJ5* mutation, and one APA without known mutation, using an anti-active β-catenin antibody (Millipore 05–655; dilution 1:1000), and anti-actin (Santa Cruz-1616) as loading control.

### Semiquantitative RT-PCR

The expression of CYP11B2 was measured using an mRNA specific custom assay (ThermoFisher Scientific, Waltham, USA)^38^. AXIN2 mRNA expression was quantified using SYBR green with FW primer: 5′-GGTCCACGGAAACTGTTGACA and RW: 5′-GGCTGGTGCAAAGACATAGCC. An mRNA specific GAPDH assay was used as internal control (4352934, ThermoFisher Scientific, Waltham, USA). The expression was measured in duplicates and a relative difference was obtained using the 2^(−ΔΔCT)^ method^53^.

### Chemiluminescent immunoassay

Sections (20 μm) of frozen tumor tissue from three tumors with *CTNNB1* mutations, six with *KCNJ5* mutation and one NHPA, were weighed with a highly sensitive scale (±0.01 mg) and homogenized in filtered water. The lysate was again weighed and a concentration of mg of tumor tissue per mg of water was calculated. The samples were then centrifuged for 20 minutes at 20000 RPM. The obtained supernatant was diluted 1:10 and subjected to an aldosterone measurement using a chemiluminescent immunoassay (DiaSorin, Italy) used in routine clinical practice at the Department of Clinical Chemistry at Uppsala University hospital. A concentration in pmol/L was obtained and adjusted to the concentration. This gave a concentration of aldosterone/mg tissue (pmol/mg).

### Statistics

The overall group effect was analyzed using a Kruskal-Wallis test. If significant group effects were present the overall analysis was followed by post-hoc comparisons between groups using a Mann-Whitney U-test with Bonferroni correction. Categorical data were analyzed by Chi-squared tests. Data are expressed as either mean ± SD or median and range for non-parametric data. For statistical analysis SPSS 22 (IBM, NY, USA) was used.

## Additional Information

**How to cite this article**: Åkerström, T. *et al.* Activating mutations in *CTNNB1* in aldosterone producing adenomas. *Sci. Rep.* **6**, 19546; doi: 10.1038/srep19546 (2016).

## Supplementary Material

Supplementary data
