# Supplementary material for: Activating mutations in CTNNB1 in aldosterone producing adenomas
Source: Sci Rep. 2016 Jan 27;6:19546. doi: 10.1038/srep19546 (PMC4728393; doi:10.1038/srep19546)
Supplement: Supplementary data [file srep19546-s1.pdf]

Activating mutations in *CTNNB1* in aldosterone producing adenomas

Tobias Åkerström[1], Rajani Maharjan[1], Holger Sven Willenberg[2], Kenko Cupisti[3], Julian Ip[4], Ana Moser[5], Peter Stålberg[1], Bruce Robinson[4], K. Alexander Iwen[5], Henning Dralle[6], Martin K. Walz[7], Hendrik Lehnert[5], Stan Sidhu[4], Celso Gomez-Sanchez[8], Per Hellman[1], and Peyman Björklund[1*].

[1]Department of Surgical Sciences, Uppsala University, Uppsala, Sweden.

[2]Department of Endocrinology and Metabolism, Rostock University Medical Center, Germany.

[3]General, Visceral and Pediatric Surgery University Hospital Düsseldorf, Düsseldorf, Germany.

[4]University of Sydney, Endocrine Surgical Unit and Cancer Genetics, Hormones and Cancer Group , Kolling Institute of Medical Research, Royal North Shore Hospital, Sydney, Australia .

[5]Department of Medicine I, University of Lübeck, University Hospital Schleswig-Holstein, Lübeck, Germany

[6]Department of General, Visceral and Vascular Surgery, University Hospital, University of Halle-Wittenberg, Halle/Saale, Germany. Sweden.

[7]Klinik für Chirurgie und Zentrum für Minimal Invasive Chirurgie, Kliniken Essen-Mitte, Essen, Germany.

[8]Endocrine Section, Department of Medicine, G.V. (Sonny) Montgomery VA Medical Center and University of Mississippi Medical Center, Jackson, USA.

*Corresponding author: Peyman Björklund, Experimental Surgery Group, Uppsala University, Akademiska sjukhuset, ing 70 FOA2, 75185 Uppsala, Sweden. Phone: +46186114911 Fax: +46186114911 Email: peyman.bjorklund@surgsci.uu.se

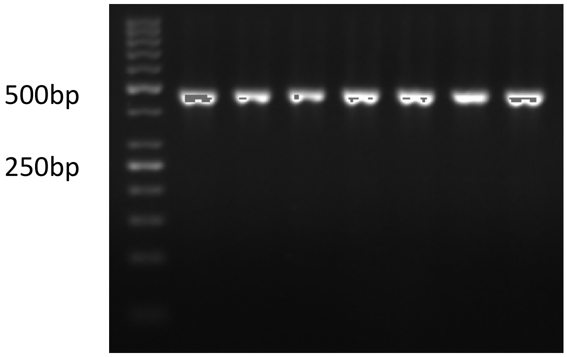

Supplementary Fig. S1. Investigation of large exon 3 *CTNNB1* deletions.

RT-PCR on cDNA from non-mutated APA samples using mRNA specific primers. Expected size of amplicon 464bp.

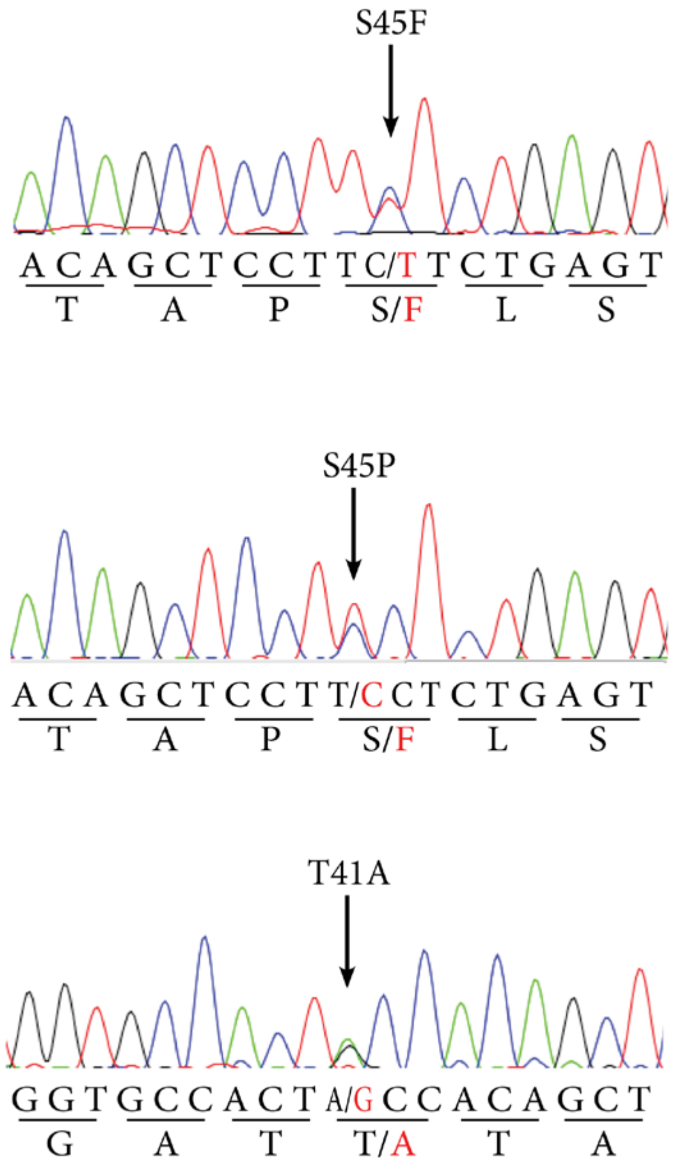

Supplementary Figure S2. Observed missense mutations in *CTNNB1*.

Chromatograms showing the different somatic *CTNNB1* mutations in APAs.

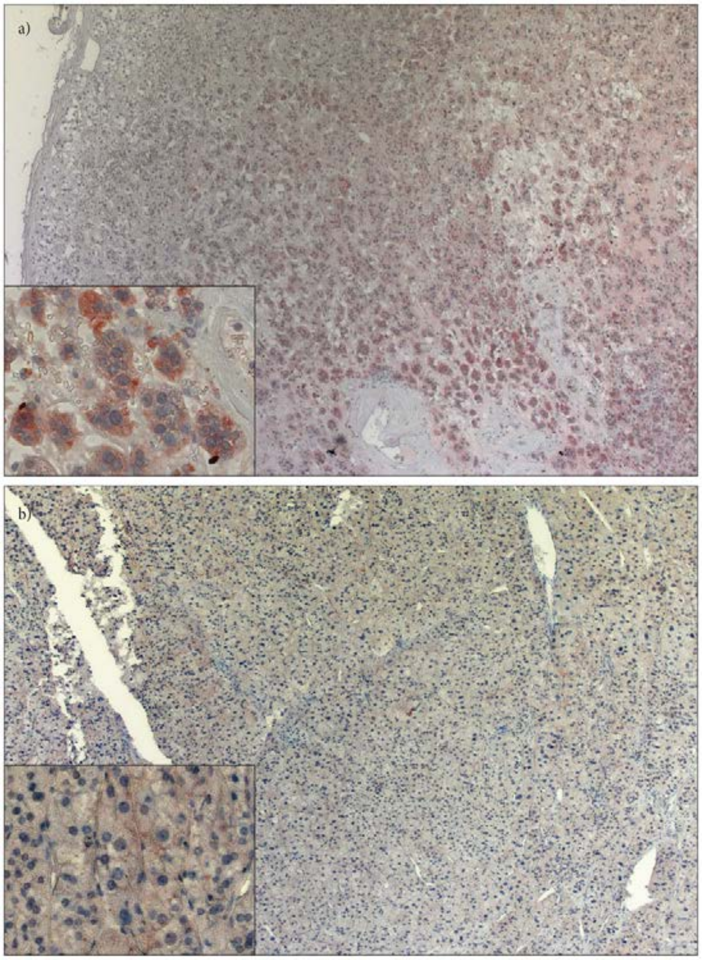

Supplementary Fig. S3. **Immunohistochemical analysis of β-catenin.**

APA with a *KCNJ5* mutation showing an area of ZG-like cells with strong cytoplasmic staining (a). APA with a *KCNJ5* mutation with mostly ZF-like cells and membranous expression (b).

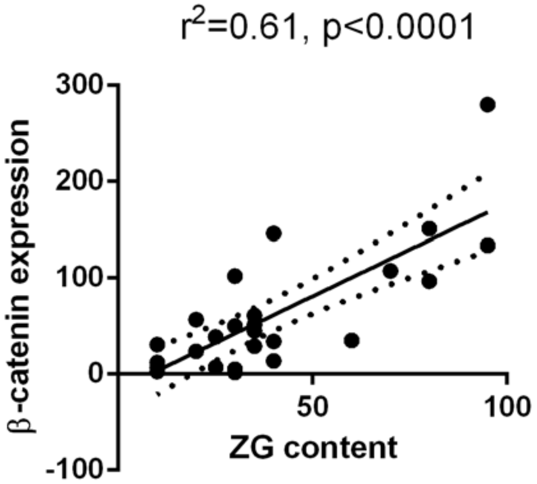

Supplementary Fig. S4. β-catenin expression and ZG-like cell content.

Correlation of ZG-like cell content and β-catenin expression. ZG=Zona Glomerulsosa

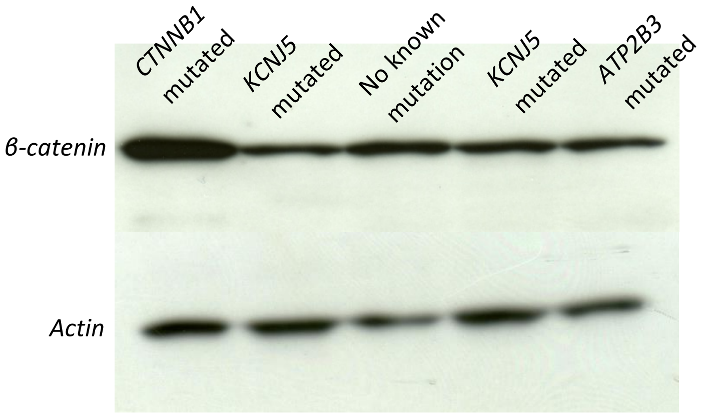

Supplementary Figure S5. **Western blot analysis of antibody used for immunohistochemistry.**

Western blot using a goat polyclonal anti β-catenin in one tumor with *CTNNB1* mutation (p.Ser45Pro) and four without *CTNNB1* mutation. Anti-actin as loading control.

Cohort	No. of APAs	*CTNNB1*
**Sweden**	41	3 (7.3%)
**Germany**	123	5 (4.1%)
**Australia**	34	2 (5.9%)
**Total:**	**198**	**10 (5.1%)**

Supplementary Table S1. *CTNNB1* mutations found in different cohorts.

																	T41				S45											
NP_001091679.1 (H. Sapiens)	**26-**	Q	Q	S	Y	L	-	D	S	G	I	H	S	G	A	T	T	T	A	P	S	L	S	G	K	G	N	P	E	E	E	**-55**
XP_001138023.1 (P.Troglodytes)	**26-**	Q	Q	S	Y	L	-	D	S	G	I	H	S	G	A	T	T	T	A	P	S	L	S	G	K	G	N	P	E	E	E	**-55**
NP_001131124.1 (C. Lupus)	**26-**	Q	Q	S	Y	L	-	D	S	G	I	H	S	G	A	T	T	T	A	P	S	L	S	G	K	G	N	P	E	E	E	**-55**
NP_445809.2 (R. Norvegicus)	**26-**	Q	Q	S	Y	L	-	D	S	G	I	H	S	G	A	T	T	T	A	P	S	L	S	G	K	G	N	P	E	E	E	**-55**
NP_001159374.1 (M. Musculus)	**26-**	Q	Q	S	Y	L	-	D	S	G	I	H	S	G	A	T	T	T	A	P	S	L	S	G	K	G	N	P	E	E	E	**-55**
NP_001069609.1 (B. Taurus)	**26-**	Q	Q	S	Y	L	-	D	S	G	I	H	S	G	A	T	T	T	A	P	S	L	S	G	K	G	N	P	E	E	E	**-55**
NP_990412.1 (G. Gallus)	**26-**	Q	Q	S	Y	L	-	D	S	G	I	H	S	G	A	T	T	T	A	P	S	L	S	G	K	G	N	P	E	E	E	**-55**
NP_571134.2 (D.Rerio)	**26-**	Q	Q	S	Y	L	-	D	S	G	I	H	S	G	A	T	T	T	A	P	S	L	S	G	K	G	N	P	E	D	D	**-55**
XP_309245.4 (A. Gambiae)	**36-**	Q	N	S	Y	L	G	D	S	G	I	H	S	G	A	V	T	Q	V	P	S	L	S	G	K	-	-	-	D	D	D	**-62**
NP_996328.1 (D. Melanogaster)	**26-**	Q	N	S	Y	L	G	D	S	G	I	H	S	G	A	V	T	Q	V	P	S	L	S	G	K	-	-	-	E	D	E	**-52**

Supplementary Table S2. Conservation of the mutated amino acid residues in *CTNNB1*.

No.	*CTNNB1* mutation	β-catenin IHC	CYP11B2 IHC (SCORE)	CYP11B1 IHC (SCORE)	Cell morphology	Gender 1=Male, 0=female	Age at op.	Adenoma size (mm)	Aldosterone pmol/L	Renin	Hypokalemia 1=yes, 0=no	Subtype classification	Postoperative outcome
1	S45P	Cytoplasmic	375	0.67	ZF	0	39	40	1168	<1	1	CT	Normotensive, eukalemic
2	S45P	Cytoplasmic/nuclear	277	0.22	ZG/ZF	0	51	13	790	7.0	1	AVS	na
3	S45P	Cytoplasmic	300	101	ZF	0	51	23	1567	1.0	1	na	na
4	S45F	Cytoplasmic/heterogenous	28.7	291	ZF>ZG	0	26	39	477	2.3	1	na	na
5	T41A	na	na	na	na	1	35	25	2441	4.0	na	CT	na
6	S45P	na	na	na	na	1	66	45	na	na	na	MRI	na
7	S45P	Cytoplasmic/nuclear	30.0	213	ZG>ZF	1	30	10	2011	1.5	1	CT/AVS	Normotensive, eukalemic
8	S45P	Membranous/nuclear	18.3	202	ZG/ZF	1	34	20	2808	2.1	1	CT/AVS	Normotensive, eukalemic
9	S45P	Cytoplasmic/heterogenous	0.97	326	ZF>ZG	0	76	30	2300	1.6	1	CT/AVS	Normotensive, eukalemic
10	S45P	Cytoplasmic/nuclear	244	0.58	ZG>ZF	0	32	15	2520	<1	1	CT/AVS	Normotensive, eukalemic

Supplementary Table S3. Characteristics of patients with *CTNNB1* mutated tumors.

Tumor size was measured either at surgery or by the pathologist. Workup describes the lateralization diagnostics made before surgery. *Plasma renin concentration measured in mU/L. Numbers in parenthesis shows postoperative values. CYP11B2 are graded from 0-400 using a modified H-score. ARR= Aldosterone to renin ratio, AVS=Adrenal vein sampling, CT=Computer tomography. na=not available. ZG=Zona Glomerulosa. ZF=Zona Fasciculata.

Patient no.	Mutation	H-SCORE	ZF %	ZG %
**1**	Non-mutated	61,11	65	35
**2**	*KCNJ5*	23,96	80	20
**3**	*KCNJ5*	13,75	60	40
**4**	Non-mutated	51,39	65	35
**5**	*KCNJ5*	50,00	70	30
**6**	Non-mutated	35,00	40	60
**7**	*KCNJ5*	107,43	30	70
**8**	*KCNJ5*	4,38	90	10
**9**	Non-mutated	29,03	65	35
**10**	*KCNJ5*	2,67	90	10
**11**	Non-mutated	45,00	65	35
**12**	Non-mutated	12,00	90	10
**13**	*KCNJ5*	1,94	70	30
**14**	Non-mutated	30,69	90	10
**15**	Non-mutated	56,88	80	20
**16**	Non-mutated	280,00	5	95
**17**	Non-mutated	7,08	75	25
**18**	Non-mutated	96,67	20	80
**19**	Non-mutated	133,68	5	95
**20**	*CTNNB1*	38,96	75	25
**21**	*KCNJ5*	34,31	60	40
**22**	*KCNJ5*	6,67	90	10
**23**	*KCNJ5*	102,22	70	30
**24**	*KCNJ5*	5,15	70	30
**25**	*CTNNB1*	151,04	20	80
**26**	*KCNJ5*	146,25	60	40

Supplementary Table S4. Immunohistochemical analysis of β-catenin in APAs.

Scoring of expression and amount of ZF-, and ZG like cells. ZF=Zona Fasciculata, ZG=Zona Glomerulosa

	(A)<br>WT<br>(n=84)	(B)<br>*KCNJ5*<br>(n=92)	(C)<br>*CTNNB1*<br>(n=10)	Between groups comparison (A-C)<br>(*P*)	A vs C<br>(*P*)	B vs C<br>(*P*)
**Age at operation**	53.5 (±7.8)	45.6 (±11.0)	37.0 (26-76)	0.000018	ns	ns
**Gender, Female in %**	40	83	60	$4.4e^{-8}$	ns*	ns*
**Tumor size (mm)**	14.7 (±7.9)	18.4 (±6.8)	24 (10-45)	0.000024	0.01	ns
**Aldosterone (pmol/L)**	1137 (±789)	1377<br>(±1694)	1567 (477-2520)	ns	ns	ns

Supplementary Table S5. Clinical data analysis.

Values are presented as mean ±SD for parametric data, and median and range for non-parametric data. Statistical analysis was performed by between groups tests (A, B, C) using a Kruskal-Wallis test. Post hoc comparison (A vs C and B vs C) using a multiple comparison corrected Mann-Whitney U-test with p<0.05/4 as cutoff point. *Chi squared test. WT= No mutation in *KCNJ5*, *ATP1A1*, *ATP2B3*, *CACNA1D* or *CTNNB1*.
